# Supplementary material for: Healthcare Expenditures among the Elderly in China: The Role of Catastrophic Medical Insurance
Source: Int J Environ Res Public Health. 2022 Nov 2;19(21):14313. doi: 10.3390/ijerph192114313 (PMC9656772; doi:10.3390/ijerph192114313)
Supplement: Supplementary file 1 [file ijerph-19-14313-s001.zip › ijerph-1937224-supplementary.pdf]

Table S1. The impact of CMI on healthcare expenditures among the Chinese elderly (DID model).

Variables	(1) Logarithm Total Medical Expenditure	(2) Logarithm Total OOP Expenditure	(3) Logarithm Inpatient Expenditure	(4) Logarithm Inpatient OOP Expenditure
$treat_{ipt} \times Post_{it}$	0.779***	0.681***	0.669***	0.751***
	(0.260)	(0.177)	(0.175)	(0.211)
$treat_{ipt}$	-1.248***	-1.305***	0.459	0.372
	(0.324)	(0.337)	(0.435)	(0.309)
$Post_{it}$	-	-	-	-
Age	-0.005	-0.010	0.007	-0.002
	(0.006)	(0.006)	(0.006)	(0.006)
Female (Ref: Male)	0.155*	0.253***	0.038	0.138
	(0.092)	(0.093)	(0.098)	(0.084)
Years of education	0.013	-0.011	0.012	0.006
	(0.014)	(0.015)	(0.016)	(0.013)
Urban or town (Ref: Rural)	0.262	-0.011	-0.211	-0.211
	(0.161)	(0.166)	(0.202)	(0.186)
Married (Ref: No)	0.037	0.040	0.104	0.097
	(0.104)	(0.110)	(0.117)	(0.085)
Number of Children alive	0.031*	0.054***	-0.026	-0.021
	(0.016)	(0.017)	(0.024)	(0.022)
Live alone (Ref: No)	-0.228**	-0.118	-0.090	0.001
	(0.097)	(0.110)	(0.123)	(0.106)
URBMI (Ref: NCMS)	0.300**	-0.080	0.019	-0.020
	(0.143)	(0.167)	(0.181)	(0.166)
Logarithm of household income per capita	-0.023	-0.049	0.043	0.037
	(0.028)	(0.031)	(0.039)	(0.038)
Self-rated Health	0.483***	0.494***	0.278***	0.255***
	(0.049)	(0.045)	(0.056)	(0.053)
Number of chronic diseases	-0.385	-0.294	0.431	-0.086
	(0.464)	(0.590)	(0.635)	(0.604)
Serious Illness in the past 2 years (Ref: No)	2.145***	2.069***	5.000***	4.269***
	(0.093)	(0.110)	(0.164)	(0.192)
Hypertension	0.921*	0.821	-0.350	0.174
	(0.465)	(0.608)	(0.621)	(0.600)
Diabetes	1.021**	0.737	-0.465	0.217
	(0.488)	(0.577)	(0.689)	(0.668)
Heart Disease	0.927**	0.763	-0.171	0.207

	(0.444)	(0.580)	(0.589)	(0.597)
Stroke or CVD	0.668	0.339	-0.290	0.187
	(0.507)	(0.616)	(0.665)	(0.616)
Bronchitis	0.848*	0.693	-0.275	0.207
	(0.457)	(0.590)	(0.636)	(0.639)
Tuberculosis	0.699	0.787	-0.788	-0.171
	(0.686)	(0.718)	(0.901)	(0.848)
Cataracts	0.527	0.441	-0.415	0.122
	(0.507)	(0.620)	(0.700)	(0.639)
Glaucoma	0.102	-0.154	-0.335	0.155
	(0.612)	(0.765)	(0.684)	(0.700)
Cancer	2.305***	1.906**	1.084	0.969
	(0.521)	(0.761)	(1.052)	(1.079)
Prostate Tumor	0.661	0.426	-0.622	0.072
	(0.482)	(0.572)	(0.728)	(0.637)
Gastric	0.890*	0.856	-0.658	0.045
	(0.504)	(0.629)	(0.757)	(0.751)
Parkinson	1.291*	0.853	0.516	0.518
	(0.656)	(0.789)	(0.799)	(0.891)
Bedsore	-1.406	-1.124	-0.355	0.252
	(1.107)	(1.098)	(0.930)	(0.948)
Arthritis	0.931**	0.893	-0.475	0.038
	(0.456)	(0.584)	(0.627)	(0.612)
Dementia	-0.247	-0.412	-1.272	-0.413
	(0.637)	(0.900)	(1.031)	(0.938)
Epilepsy	1.596***	1.984***	0.565	1.916***
	(0.564)	(0.597)	(0.630)	(0.661)
Cholecystitis	0.606	0.383	-0.353	0.242
	(0.464)	(0.595)	(0.641)	(0.596)
Blood Disease	0.494	0.215	0.112	0.402
	(0.506)	(0.613)	(0.748)	(0.686)
Chronic Nephritis	0.255	-0.154	0.110	0.937
	(0.566)	(0.782)	(0.976)	(0.939)
Galactophore	1.500	1.424	2.495	2.960**
	(0.957)	(1.120)	(1.510)	(1.300)
Uterine Tumor	1.233	1.207	-0.169	0.464
	(0.959)	(1.045)	(1.270)	(1.334)
Hepatitis	-	-	-	-
Year = 2011	-0.080	-0.126		
	(0.121)	(0.098)		
Year = 2014	0.188	-0.118	0.147	-0.061
	(0.120)	(0.116)	(0.106)	(0.105)
Province: Tianjing	-0.404	-0.007	0.427	0.648*

	(0.484)	(0.556)	(0.418)	(0.352)
Province: Hebei	-0.479	-0.452	-0.083	0.043
	(0.538)	(0.418)	(0.410)	(0.280)
Province: Shanxi	-2.012***	-2.102***	0.568	0.640**
	(0.358)	(0.412)	(0.410)	(0.283)
Province: Liaoning	-0.867**	-1.221***	-0.170	-0.626***
	(0.329)	(0.255)	(0.126)	(0.144)
Province: Jilin	-0.513	0.020	0.324	0.575
	(0.560)	(0.579)	(0.505)	(0.428)
Province: Helongjiang	-1.619***	-1.602***	0.070	0.287
	(0.281)	(0.279)	(0.396)	(0.267)
Province: Shanghai	-1.386**	-1.797***	-0.452	-0.213
	(0.572)	(0.419)	(0.431)	(0.291)
Province: Jiangsu	-0.926***	-1.338***	-0.046	0.061
	(0.289)	(0.293)	(0.448)	(0.353)
Province: Zhejiang	0.539*	0.457*	-0.775***	-0.706***
	(0.304)	(0.257)	(0.130)	(0.170)
Province: Anhui	-1.527***	-1.549***	0.522	0.723**
	(0.327)	(0.362)	(0.517)	(0.356)
Province: Fujian	0.340	0.577*	-0.928***	-0.654***
	(0.260)	(0.293)	(0.150)	(0.164)
Province: Jiangxi	-0.966***	-1.247***	-0.171	-0.085
	(0.262)	(0.333)	(0.445)	(0.336)
Province: Shangdong	-1.452***	-1.516***	0.231	0.156
	(0.302)	(0.294)	(0.417)	(0.276)
Province: Henan	-0.958***	-1.086***	0.326	0.163
	(0.249)	(0.296)	(0.425)	(0.408)
Province: Hubei	-0.393	-0.386	-0.966***	-1.008***
	(0.310)	(0.281)	(0.215)	(0.317)
Province: Hunan	-1.484***	-1.496***	0.033	0.010
	(0.344)	(0.390)	(0.467)	(0.395)
Province: Guangdong	-1.595***	-1.522***	0.113	0.109
	(0.360)	(0.377)	(0.428)	(0.283)
Province: Guangxi	-1.705***	-1.949***	0.081	0.068
	(0.314)	(0.297)	(0.473)	(0.331)
Province: Hainan	-0.977***	-1.013**	0.315	-0.317
	(0.366)	(0.472)	(0.441)	(0.558)
Province: Chongqing	-	-	-	-
Province: Sichuan	-1.125***	-1.143***	0.443	0.425
	(0.279)	(0.295)	(0.434)	(0.279)
Province: Shaanxi	-0.578**	-0.960***	0.102	0.148
	(0.260)	(0.267)	(0.509)	(0.323)
Constant	4.772***	5.146***	-1.114	-0.400

	(0.646)	(0.619)	(0.690)	(0.611)
Observations	4,629	4,629	3,086	3,086
Adjusted R-squared	0.246	0.203	0.405	0.368

Note: *** P<0.01 ** P<0.05 * P<0.1; Robust Standard errors clustered in province level are reported in the Parentheses.

Table S2. The impact of CMI on healthcare expenditures among the Chinese elderly (Fixed effects model)

Variables	(1) Logarithm Total Medical Expenditure	(2) Logarithm Total OOP Expenditure	(3) Logarithm Inpatient Expenditure	(4) Logarithm Inpatient OOP Expenditure
$treat_{ipt} \times Post_{it}$	0.776**	0.649***	0.577**	0.625**
	(0.310)	(0.172)	(0.225)	(0.237)
Age	0.353	0.398*	0.411**	0.529**
	(0.209)	(0.209)	(0.147)	(0.203)
Female (Ref: Male)	-	-	-	-
Years of education	-	-	-	-
Urban or town (Ref: Rural)	0.122	-0.128	0.166	0.226
	(0.325)	(0.295)	(0.449)	(0.409)
Married (Ref: No)	-0.157	-0.047	0.348	-0.017
	(0.176)	(0.178)	(0.339)	(0.309)
Number of Children alive	-0.005	-0.002	-0.076**	-0.162***
	(0.033)	(0.030)	(0.033)	(0.055)
Live alone (Ref: No)	-0.403**	-0.196	-0.105	0.021
	(0.165)	(0.181)	(0.338)	(0.312)
URBMI (Ref: NCMS)	0.303**	0.140	0.124	0.101
	(0.132)	(0.117)	(0.247)	(0.262)
Logarithm of household income per capita	-0.016	-0.031	0.001	-0.066
	(0.035)	(0.031)	(0.058)	(0.068)
Self-rated Health	0.393***	0.411***	0.187*	0.242**
	(0.062)	(0.058)	(0.097)	(0.091)
Number of chronic diseases	1.629***	2.927*	1.789***	1.756***
	(0.318)	(1.450)	(0.563)	(0.510)
Serious Illness in the past 2 years (Ref: No)	1.888***	1.872***	4.396***	3.799***
	(0.181)	(0.130)	(0.182)	(0.186)
Hypertension	-1.242***	-2.536	-1.713**	-1.797***
	(0.394)	(1.524)	(0.639)	(0.537)
Diabetes	-0.684	-2.253	-1.507	-1.268

	(0.492)	(1.508)	(0.878)	(0.843)
Heart Disease	-1.266***	-2.465	-1.539***	-1.589***
	(0.360)	(1.503)	(0.543)	(0.531)
Stroke or CVD	-1.347***	-2.808*	-1.517**	-1.568**
	(0.315)	(1.444)	(0.631)	(0.608)
Bronchitis	-1.381***	-2.614*	-1.326**	-1.251**
	(0.370)	(1.484)	(0.616)	(0.601)
Tuberculosis	-1.822**	-3.061**	-2.936***	-2.899***
	(0.765)	(1.246)	(0.920)	(0.986)
Cataracts	-1.508***	-2.730*	-2.184***	-1.971***
	(0.357)	(1.435)	(0.609)	(0.552)
Glaucoma	-0.731*	-2.194	-1.301	-0.902
	(0.380)	(1.375)	(1.309)	(1.202)
Cancer	0.174	-1.368	-0.676	-1.242
	(0.656)	(2.100)	(1.824)	(1.691)
Prostate Tumor	-1.716***	-2.895*	-1.849**	-1.653**
	(0.396)	(1.442)	(0.857)	(0.717)
Gastric	-1.347***	-2.551*	-1.920***	-1.740***
	(0.342)	(1.451)	(0.613)	(0.610)
Parkinson	-0.495	-1.831	0.137	-0.662
	(0.609)	(1.556)	(1.788)	(1.586)
Bedsore	-4.299***	-5.409**	-2.786	-2.576
	(1.187)	(2.116)	(1.683)	(1.621)
Arthritis	-1.145***	-2.425	-2.042***	-1.986***
	(0.336)	(1.446)	(0.562)	(0.440)
Dementia	-3.106***	-4.648**	-1.802	-1.637
	(0.791)	(1.736)	(1.350)	(1.254)
Epilepsy	-	-	-	-
Cholecystitis	-1.441***	-2.912*	-1.207*	-1.093
	(0.351)	(1.466)	(0.699)	(0.638)
Blood Disease	-1.640***	-2.912*	-0.863	-0.965
	(0.427)	(1.584)	(0.773)	(0.803)
Chronic Nephritis	-1.935***	-3.369**	-0.425	-0.476
	(0.528)	(1.286)	(1.057)	(1.005)
Galactophore	-1.292	-2.723	-1.325	-1.707
	(1.190)	(1.819)	(1.198)	(1.324)
Uterine Tumor	-0.641	-1.970	-0.925	-0.882
	(0.782)	(1.611)	(1.241)	(1.352)
Hepatitis	-2.123***	-3.283*	-1.235	-1.420*
	(0.561)	(1.671)	(0.856)	(0.772)
Year = 2011	-1.115	-1.354*		
	(0.707)	(0.695)		
Year = 2014	-1.798	-2.441*	-0.877**	-1.401**

	(1.261)	(1.225)	(0.351)	(0.540)
Observations	4,629	4,629	3,086	3,086
Number of id	1,543	1,543	1,543	1,543
Adjusted R-squared	0.149	0.126	0.312	0.296

Note: *** P<0.01 ** P<0.05 * P<0.1; Robust Standard errors clustered in province level are reported in the Parentheses.

Table S3. The impact of CMI on healthcare expenditures among the Chinese elderly (Fixed effects model and rural sample)

Variables	(1) Logarithm Total Medical Expenditure	(2) Logarithm Total OOP Expenditure	(3) Logarithm Inpatient Expenditure	(4) Logarithm Inpatient OOP Expenditure
$treat_{ipt} \times Post_{it}$	0.810***	0.641***	0.607**	0.704***
	(0.214)	(0.220)	(0.274)	(0.252)
Age	0.331*	0.396**	0.392	0.549**
	(0.170)	(0.174)	(0.249)	(0.224)
Female (Ref: Male)	-	-	-	-
Years of education	-	-	-	-
Urban or town (Ref: Rural)	-	-	-	-
Married (Ref: No)	-0.139	-0.037	0.189	-0.080
	(0.245)	(0.255)	(0.421)	(0.393)
Number of Children alive	-0.007	-0.005	-0.033	-0.130***
	(0.035)	(0.035)	(0.043)	(0.043)
Live alone (Ref: No)	-0.354*	-0.173	-0.307	-0.140
	(0.195)	(0.194)	(0.304)	(0.279)
URBMI (Ref: NCMS)	0.496**	0.202	0.624*	0.504
	(0.196)	(0.197)	(0.370)	(0.330)
Logarithm of household income per capita	0.001	-0.011	-0.004	-0.074
	(0.043)	(0.045)	(0.061)	(0.064)
Self-rated Health	0.383***	0.421***	0.204*	0.266***
	(0.063)	(0.064)	(0.106)	(0.099)
Number of chronic	-0.476	-0.010	0.451	0.498

diseases				
	(0.791)	(0.745)	(0.834)	(0.697)
Serious Illness in the past 2 years (Ref: No)	1.914***	1.807***	4.389***	3.756***
	(0.141)	(0.143)	(0.261)	(0.238)
Hypertension	0.699	0.278	-0.537	-0.603
	(0.813)	(0.766)	(0.874)	(0.731)
Diabetes	1.483*	0.632	0.458	0.564
	(0.880)	(0.855)	(1.069)	(0.942)
Heart Disease	1.033	0.545	-0.079	-0.386
	(0.818)	(0.781)	(0.932)	(0.781)
Stroke or CVD	0.789	-0.046	-0.007	-0.365
	(0.819)	(0.776)	(0.983)	(0.821)
Bronchitis	0.768	0.286	-0.091	-0.071
	(0.801)	(0.744)	(0.864)	(0.735)
Tuberculosis	-0.022	-0.621	-1.775	-1.680
	(1.141)	(1.087)	(1.242)	(1.095)
Cataracts	0.521	0.288	-0.865	-0.747
	(0.798)	(0.753)	(0.899)	(0.747)
Glaucoma	1.453*	0.665	-0.154	-0.165
	(0.858)	(0.835)	(1.343)	(1.190)
Cancer	3.384***	2.794**	2.669	2.610
	(1.173)	(1.091)	(1.852)	(1.767)
Prostate Tumor	0.557	0.322	-0.479	-0.459
	(0.829)	(0.790)	(0.920)	(0.789)
Gastric	0.807	0.375	-0.676	-0.611
	(0.809)	(0.770)	(0.944)	(0.813)
Parkinson	2.102*	1.378	2.976*	1.571
	(1.140)	(0.980)	(1.601)	(1.508)
Bedsore	-2.565*	-2.898**	-1.322	-1.115
	(1.364)	(1.222)	(2.537)	(2.364)
Arthritis	1.014	0.534	-0.669	-0.741
	(0.806)	(0.761)	(0.868)	(0.734)
Dementia	-0.740	-1.618	-0.429	-0.380
	(1.090)	(1.088)	(1.214)	(1.064)
Epilepsy	1.888**	0.902		
	(0.801)	(0.758)		
Cholecystitis	0.611	0.061	0.200	0.130
	(0.857)	(0.816)	(0.965)	(0.835)
Blood Disease	0.699	0.238	0.351	0.232
	(0.855)	(0.820)	(0.987)	(0.864)
Chronic Nephritis	-0.337	-0.820	0.213	0.399
	(0.975)	(1.078)	(1.454)	(1.301)
Galactophore	0.170	-0.708	-0.696	-1.460

	(1.208)	(1.245)	(2.240)	(2.241)
Uterine Tumor	0.245	0.338	-0.054	-0.082
	(1.357)	(1.429)	(1.813)	(1.802)
Hepatitis	-	-	-	-
Year = 2011	-0.985*	-1.346**		
	(0.521)	(0.533)		
Year = 2014	-1.666*	-2.492**	-0.845	-1.529**
	(0.985)	(1.010)	(0.689)	(0.617)
Observations	3,944	3,944	2,600	2,600
Number of id	1,386	1,386	1,338	1,338
Adjusted R-squared	0.148	0.119	0.314	0.291

Note: *** P<0.01 ** P<0.05 * P<0.1; Robust Standard errors clustered in province level are reported in the Parentheses.

Table S4. The impact of CMI on healthcare expenditures among the Chinese elderly (Fixed effects model and urban or town sample)

Variables	(1) Logarithm Total Medical Expenditure	(2) Logarithm Total OOP Expenditure	(3) Logarithm Inpatient Expenditure	(4) Logarithm Inpatient OOP Expenditure
$treat_{ipt} \times Post_{it}$	0.698	0.380	-0.232	-0.440
	(0.518)	(0.536)	(0.593)	(0.514)
Age	0.080	0.090	0.429	0.520
	(0.475)	(0.470)	(0.685)	(0.639)
Female (Ref: Male)	-	-	-	-
Years of education	-	-	-	-
Urban or town (Ref: Rural)	-	-	-	-
Married (Ref: No)	0.107	0.211	2.448*	0.520
	(0.601)	(0.638)	(1.438)	(0.687)
Number of Children alive	-0.120	-0.132	-0.371*	-0.340*
	(0.089)	(0.106)	(0.195)	(0.188)
Live alone (Ref: No)	-0.410	-0.066	0.264	0.406
	(0.546)	(0.590)	(1.000)	(0.848)
URBMI (Ref: NCMS)	-0.056	0.087	-0.500	-0.524
	(0.350)	(0.374)	(0.491)	(0.442)

Logarithm of household income per capita	-0.146	-0.305*	-0.079	-0.062
	(0.134)	(0.175)	(0.185)	(0.174)
Self-rated Health	0.545***	0.370**	0.192	0.114
	(0.184)	(0.187)	(0.328)	(0.293)
Number of chronic diseases	-1.251	-1.948**	1.846	0.532
	(1.104)	(0.961)	(1.379)	(1.419)
Serious Illness in the past 2 years (Ref: No)	1.679***	1.764***	4.294***	3.879***
	(0.304)	(0.330)	(0.596)	(0.512)
Hypertension	2.564**	3.096***	-1.267	-0.637
	(1.224)	(1.128)	(1.703)	(1.669)
Diabetes	2.298	2.813**	-3.217**	-2.055
	(1.464)	(1.344)	(1.615)	(1.568)
Heart Disease	0.975	2.309**	-1.393	0.363
	(1.229)	(1.083)	(1.522)	(1.569)
Stroke or CVD	1.842	3.398***	-1.640	-0.017
	(1.370)	(1.131)	(1.814)	(1.771)
Bronchitis	1.343	2.425**	-0.516	0.210
	(1.194)	(1.074)	(1.749)	(1.697)
Tuberculosis	3.962**	3.515*	-4.385**	-4.647***
	(1.655)	(1.995)	(1.961)	(1.699)
Cataracts	1.661	2.007*	-2.772*	-1.479
	(1.314)	(1.124)	(1.641)	(1.642)
Glaucoma	2.040	3.101**	-0.329	4.622*
	(1.449)	(1.461)	(1.771)	(2.425)
Cancer	1.223	1.423	-6.201**	-5.953***
	(1.261)	(1.880)	(2.545)	(2.188)
Prostate Tumor	0.826	1.294	-2.715*	-0.926
	(1.277)	(1.119)	(1.627)	(1.600)
Gastric	0.966	2.581**	-0.540	1.166
	(1.276)	(1.178)	(1.608)	(1.624)
Parkinson	1.419	2.965**	3.531	4.230
	(1.937)	(1.445)	(4.856)	(3.795)
Bedsore	-	-	-	-
Arthritis	1.400	2.355**	-2.173	-0.451
	(1.163)	(0.986)	(1.629)	(1.677)
Dementia	-1.299	-1.059	-4.916	-3.511
	(2.713)	(2.260)	(4.758)	(3.671)
Epilepsy	3.221**	7.470***	-0.353	1.159
	(1.606)	(1.418)	(2.122)	(1.979)
Cholecystitis	1.837	1.792	-2.055	-0.265
	(1.306)	(1.275)	(1.591)	(1.500)
Blood Disease	0.847	1.366	-1.078	0.440
	(1.172)	(1.115)	(1.564)	(1.555)

Chronic Nephritis	0.908	1.233	0.771	-0.008
	(1.305)	(1.549)	(3.227)	(2.674)
Galactophore	3.436	7.184**	-1.527	3.695
	(2.529)	(3.207)	(4.600)	(3.725)
Uterine Tumor	5.256***	4.286*	-0.732	0.143
	(1.731)	(2.546)	(2.905)	(2.419)
Hepatitis	-	-	-	-
Year = 2011	-0.524	-0.253		
	(1.486)	(1.483)		
Year = 2014	-0.129	-0.131	-0.364	-0.563
	(2.818)	(2.787)	(2.001)	(1.862)
Observations	685	685	486	486
Number of id	300	300	281	281
Adjusted R-squared	0.157	0.155	0.370	0.392

Note: *** P<0.01 ** P<0.05 * P<0.1; Robust Standard errors clustered in province level are reported in the Parentheses.

Table S5. The impact of CMI on healthcare expenditures among the Chinese elderly (Fixed effects model and Low-income sample)

Variables	(1) Logarithm Total Medical Expenditure	(2) Logarithm Total OOP Expenditure	(3) Logarithm Inpatient Expenditure	(4) Logarithm Inpatient OOP Expenditure
$treat_{ipt} \times Post_{it}$	0.515*	0.301	0.575*	0.507
	(0.275)	(0.289)	(0.316)	(0.334)
Age	0.241	0.473**	0.223	0.417
	(0.210)	(0.213)	(0.212)	(0.269)
Female (Ref: Male)	-	-	-	-
Years of education	-	-	-	-
Urban or town (Ref: Rural)	0.139	0.127	-0.479	0.394
	(0.385)	(0.422)	(0.560)	(0.427)
Married (Ref: No)	-0.235	-0.064	-0.040	-0.491
	(0.296)	(0.297)	(0.536)	(0.513)
Number of Children alive	0.008	0.047	-0.021	-0.111*
	(0.038)	(0.039)	(0.033)	(0.058)
Live alone (Ref: No)	-0.426*	-0.200	-0.094	-0.009

	(0.233)	(0.233)	(0.339)	(0.248)
URBMI (Ref: NCMS)	0.252	-0.093	0.491	0.551
	(0.288)	(0.303)	(0.714)	(0.639)
Logarithm of household income per capita	-0.016	-0.028	-0.009	-0.085
	(0.054)	(0.055)	(0.072)	(0.095)
Self-rated Health	0.340***	0.382***	0.007	0.056
	(0.078)	(0.079)	(0.138)	(0.110)
Number of chronic diseases	-1.147	-0.733	2.421	2.744
	(0.831)	(0.766)	(2.862)	(2.978)
Serious Illness in the past 2 years (Ref: No)	1.924***	1.695***	3.791***	3.223***
	(0.169)	(0.176)	(0.234)	(0.278)
Hypertension	1.262	0.906	-2.587	-2.964
	(0.861)	(0.796)	(2.813)	(2.841)
Diabetes	1.986**	1.370	-1.735	-2.003
	(0.916)	(0.872)	(2.691)	(2.921)
Heart Disease	1.772**	1.500*	-2.372	-3.079
	(0.871)	(0.820)	(2.981)	(3.176)
Stroke or CVD	1.380	0.485	-1.817	-2.354
	(0.890)	(0.819)	(2.693)	(2.799)
Bronchitis	1.286	0.757	-2.292	-2.518
	(0.867)	(0.790)	(2.810)	(2.978)
Tuberculosis	0.430	-0.259	-4.606	-4.774
	(1.490)	(1.445)	(2.861)	(2.963)
Cataracts	1.121	0.783	-2.905	-3.115
	(0.848)	(0.789)	(2.936)	(2.981)
Glaucoma	2.287**	1.726**	-2.947	-3.163
	(0.919)	(0.862)	(3.382)	(3.349)
Cancer	3.634***	2.873**	4.366	4.220
	(1.195)	(1.180)	(2.808)	(3.112)
Prostate Tumor	1.728*	1.318	-2.648	-2.786
	(0.892)	(0.828)	(3.197)	(3.159)
Gastric	1.277	0.755	-2.737	-3.022
	(0.865)	(0.808)	(2.857)	(2.965)
Parkinson	2.760***	1.759*	0.973	-0.627
	(1.018)	(0.936)	(3.090)	(3.015)
Bedsore	-0.471	-1.124		
	(1.602)	(1.627)		
Arthritis	1.611*	1.183	-2.835	-3.165
	(0.848)	(0.786)	(2.704)	(2.797)
Dementia	-0.061	-0.576	-2.439	-2.628
	(1.240)	(1.196)	(2.990)	(3.103)
Epilepsy	-	-	-	-
Cholecystitis	1.438	1.380	-1.964	-2.169

	(0.958)	(0.888)	(3.134)	(3.339)
Blood Disease	1.867**	1.650*	-0.971	-1.418
	(0.943)	(0.891)	(3.262)	(3.484)
Chronic Nephritis	-0.730	-0.390	0.235	-0.238
	(1.000)	(1.039)	(3.264)	(3.373)
Galactophore	1.327	0.877	-1.210	-2.457
	(1.230)	(1.191)	(2.831)	(2.729)
Uterine Tumor	0.801	0.186	-2.927	-3.240
	(1.616)	(1.440)	(2.979)	(3.098)
Hepatitis	-	-		
Year = 2011	-0.719	-1.575**		
	(0.644)	(0.652)		
Year = 2014	-1.019	-2.775**	-0.555	-1.286*
	(1.218)	(1.231)	(0.485)	(0.741)
Observations	2,448	2,448	1,632	1,632
Number of id	816	816	816	816
Adjusted R-squared	0.149	0.110	0.247	0.234

Note: *** P<0.01 ** P<0.05 * P<0.1; Robust Standard errors clustered in province level are reported in the Parentheses.

Table S6. The impact of CMI on healthcare expenditures among the Chinese elderly (Fixed effects model and High-income sample)

Variables	(1) Logarithm Total Medical Expenditure	(2) Logarithm Total OOP Expenditure	(3) Logarithm Inpatient Expenditure	(4) Logarithm Inpatient OOP Expenditure
$treat_{ipt} \times Post_{it}$	1.046***	0.912***	0.385	0.483**
	(0.269)	(0.272)	(0.230)	(0.210)
Age	0.524**	0.372	0.621**	0.650***
	(0.228)	(0.236)	(0.223)	(0.219)
Female (Ref: Male)	-	-	-	-
Years of education	-	-	-	-
Urban or town (Ref: Rural)	0.093	-0.297	0.610	0.285
	(0.415)	(0.419)	(0.591)	(0.495)
Married (Ref: No)	-0.093	-0.040	0.649	0.407
	(0.341)	(0.379)	(0.529)	(0.511)

Number of Children alive	-0.028	-0.106	-0.172	-0.255**
	(0.065)	(0.064)	(0.104)	(0.104)
Live alone (Ref: No)	-0.365	-0.159	-0.098	0.070
	(0.269)	(0.274)	(0.585)	(0.548)
URBMI (Ref: NCMS)	0.426**	0.327	-0.046	-0.103
	(0.197)	(0.200)	(0.168)	(0.221)
Logarithm of household income per capita	-0.065	-0.108	0.104	0.081
	(0.076)	(0.077)	(0.088)	(0.070)
Self-rated Health	0.435***	0.415***	0.303***	0.361***
	(0.088)	(0.089)	(0.098)	(0.111)
Number of chronic diseases	0.057	0.022	-6.051	-5.731
	(0.940)	(1.004)	(4.132)	(4.357)
Serious Illness in the past 2 years (Ref: No)	1.822***	2.043***	4.954***	4.332***
	(0.189)	(0.188)	(0.227)	(0.201)
Hypertension	0.601	0.607	6.310	5.805
	(0.977)	(1.037)	(4.186)	(4.398)
Diabetes	1.066	0.868	6.167	6.108
	(1.106)	(1.167)	(4.545)	(4.748)
Heart Disease	0.050	0.168	6.591	6.423
	(0.983)	(1.048)	(4.273)	(4.499)
Stroke or CVD	0.291	0.441	5.942	5.595
	(0.989)	(1.052)	(4.174)	(4.447)
Bronchitis	0.272	0.564	6.910	6.576
	(0.930)	(0.992)	(4.339)	(4.496)
Tuberculosis	0.304	0.498	5.083	4.600
	(1.229)	(1.282)	(3.977)	(4.256)
Cataracts	0.233	0.334	5.744	5.632
	(0.970)	(1.020)	(4.112)	(4.369)
Glaucoma	0.456	0.190	7.803*	8.025
	(1.057)	(1.173)	(4.543)	(4.738)
Cancer	1.432	1.099	4.356	3.085
	(1.357)	(1.540)	(4.498)	(4.536)
Prostate Tumor	-0.683	-0.511	6.010	5.815
	(0.998)	(1.042)	(4.222)	(4.499)
Gastric	0.443	0.790	6.625	6.596
	(0.984)	(1.054)	(4.139)	(4.341)
Parkinson	0.319	1.399	6.277	5.600
	(1.761)	(1.445)	(6.781)	(6.342)
Bedsore	-3.333**	-2.811*	3.422	3.280
	(1.481)	(1.490)	(5.277)	(5.288)
Arthritis	0.462	0.538	5.893	5.608
	(0.975)	(1.031)	(4.322)	(4.491)
Dementia	-1.636	-2.313	6.794	6.552
	(1.500)	(1.531)	(4.040)	(4.264)

Epilepsy	1.766	3.084*	6.453	6.091
	(1.114)	(1.775)	(4.051)	(4.324)
Cholecystitis	0.070	-0.426	6.568	6.332
	(1.030)	(1.106)	(4.035)	(4.307)
Blood Disease	-0.494	-0.532	6.637	6.266
	(0.993)	(1.070)	(4.021)	(4.225)
Chronic Nephritis	0.840	0.243	7.082	6.637
	(1.094)	(1.431)	(4.429)	(4.590)
Galactophore	-1.861	-2.067		
	(2.682)	(3.384)		
Uterine Tumor	2.409*	2.504	9.658**	9.382*
	(1.438)	(1.612)	(4.374)	(4.578)
Hepatitis	-	-		
Year = 2011	-1.653**	-1.203*		
	(0.708)	(0.730)		
Year = 2014	-2.944**	-2.370*	-1.248*	-1.492**
	(1.325)	(1.371)	(0.629)	(0.637)
Observations	2,181	2,181	1,454	1,454
Number of id	727	727	727	727
Adjusted R-squared	0.154	0.151	0.416	0.407

Note: *** P<0.01 ** P<0.05 * P<0.1; Robust Standard errors clustered in province level are reported in the Parentheses.

Table S7. The impact of CMI on healthcare expenditures among the Chinese elderly (Fixed effects model and sample among those without serious illness in the past 2 years)

Variables	(1) Logarithm Total Medical Expenditure	(2) Logarithm Total OOP Expenditure	(3) Logarithm Inpatient Expenditure	(4) Logarithm Inpatient OOP Expenditure
$treat_{ipt} \times Post_{it}$	0.726***	0.534**	0.403*	0.476**
	(0.246)	(0.255)	(0.208)	(0.210)
Age	0.353*	0.314*	0.077	0.084
	(0.186)	(0.189)	(0.233)	(0.280)
Female (Ref: Male)	-	-	-	-
Years of education	-	-	-	-

Urban or town (Ref: Rural)	0.065	-0.159	0.311	0.232
	(0.397)	(0.401)	(0.313)	(0.290)
Married (Ref: No)	-0.196	0.013	0.208	0.355*
	(0.295)	(0.308)	(0.223)	(0.199)
Number of Children alive	0.010	0.017	-0.057	-0.101
	(0.044)	(0.043)	(0.054)	(0.059)
Live alone (Ref: No)	-0.457**	-0.305	-0.497	-0.469
	(0.213)	(0.209)	(0.331)	(0.316)
URBMI (Ref: NCMS)	0.429**	0.173	-0.006	0.071
	(0.211)	(0.222)	(0.377)	(0.282)
Logarithm of household income per capita	0.022	0.030	0.032	0.020
	(0.053)	(0.053)	(0.046)	(0.035)
Self-rated Health	0.430***	0.448***	0.068	0.110
	(0.073)	(0.074)	(0.090)	(0.086)
Number of chronic diseases	-0.606	-0.523	0.188	0.067
	(1.003)	(0.980)	(0.949)	(0.784)
Serious Illness in the past 2 years (Ref: No)	-	-	-	-
Hypertension	0.927	0.821	-0.102	0.036
	(1.033)	(1.009)	(1.082)	(0.882)
Diabetes	1.819	1.410	0.328	0.445
	(1.118)	(1.101)	(0.878)	(0.767)
Heart Disease	0.993	1.179	0.095	0.284
	(1.038)	(1.029)	(0.887)	(0.741)
Stroke or CVD	0.871	0.607	-0.323	-0.545
	(1.026)	(1.026)	(1.397)	(1.125)
Bronchitis	0.970	0.923	-0.174	0.122
	(1.010)	(0.969)	(1.046)	(0.910)
Tuberculosis	0.618	0.375	-1.579	-1.445
	(1.314)	(1.282)	(1.597)	(1.375)
Cataracts	0.927	0.910	-0.168	0.111
	(1.024)	(0.988)	(1.065)	(0.859)
Glaucoma	1.784	1.129	-0.289	-0.158
	(1.114)	(1.138)	(0.938)	(0.758)
Cancer	2.181	2.221	-0.558	-0.399
	(1.595)	(1.604)	(1.048)	(0.913)
Prostate Tumor	0.712	0.677	-0.501	-0.394
	(1.050)	(1.011)	(0.918)	(0.752)
Gastric	0.760	0.743	0.517	0.636
	(1.032)	(1.016)	(1.048)	(0.885)
Parkinson	0.195	0.958	2.853	3.154
	(1.317)	(1.237)	(2.711)	(2.546)
Bedsore	-2.742*	-2.665*	-2.936	-2.532

	(1.568)	(1.456)	(2.363)	(2.059)
Arthritis	1.235	1.067	-0.489	-0.441
	(1.025)	(1.002)	(0.965)	(0.806)
Dementia	-1.910	-2.655**	-0.648	-0.442
	(1.343)	(1.331)	(1.521)	(1.310)
Epilepsy	1.877*	1.431		
	(1.010)	(0.993)		
Cholecystitis	0.437	-0.047	-0.296	-0.290
	(1.097)	(1.084)	(0.670)	(0.548)
Blood Disease	0.432	0.550	0.370	0.558
	(1.090)	(1.083)	(1.063)	(0.911)
Chronic Nephritis	0.882	0.148	0.855	0.933
	(1.113)	(1.507)	(1.288)	(1.161)
Galactophore	0.497	0.409	-8.246***	-8.145***
	(4.218)	(4.688)	(1.888)	(1.589)
Uterine Tumor	3.034	2.620		
	(2.288)	(2.275)		
Hepatitis	-	-		
Year = 2011	-1.209**	-1.246**		
	(0.570)	(0.579)		
Year = 2014	-1.859*	-2.009*	-0.058	-0.203
	(1.076)	(1.093)	(0.700)	(0.836)
Observations	3,617	3,617	2,308	2,308
Number of id	1,504	1,504	1,386	1,386
Adjusted R-squared	0.059	0.045	0.031	0.045

Note: *** P<0.01 ** P<0.05 * P<0.1; Robust Standard errors clustered in province level are reported in the Parentheses.

Table S8. The impact of CMI on healthcare expenditures among the Chinese elderly (Fixed effects model and sample among those with serious illness in the past 2 years)

Variables	(1) Logarithm Total Medical Expenditure	(2) Logarithm Total OOP Expenditure	(3) Logarithm Inpatient Expenditure	(4) Logarithm Inpatient OOP Expenditure
$treat_{ipt} \times Post_{it}$	0.989**	1.297***	1.398*	1.216
	(0.448)	(0.494)	(0.737)	(0.953)

Age	-0.441	-0.293	-0.210	0.632
	(0.496)	(0.489)	(1.208)	(1.341)
Female (Ref: Male)	-	-	-	-
Years of education	-	-	-	-
Urban or town (Ref: Rural)	0.519	0.643	-0.400	0.237
	(0.661)	(0.619)	(1.806)	(1.654)
Married (Ref: No)	0.434	-0.064	2.636**	1.355*
	(0.639)	(0.535)	(1.079)	(0.730)
Number of Children alive	0.067	-0.055	-0.241	-0.452**
	(0.112)	(0.098)	(0.235)	(0.182)
Live alone (Ref: No)	-0.444	0.116	1.452	1.266*
	(0.528)	(0.599)	(0.851)	(0.698)
URBMI (Ref: NCMS)	0.066	-0.020	0.653	0.394
	(0.373)	(0.325)	(1.151)	(1.035)
Logarithm of household income per capita	0.117	0.094	0.491**	0.126
	(0.087)	(0.099)	(0.214)	(0.300)
Self-rated Health	0.175	0.231	0.432	0.603
	(0.152)	(0.153)	(0.489)	(0.392)
Number of chronic diseases	-0.471	0.475	7.585*	5.000**
	(0.783)	(1.028)	(4.030)	(1.924)
Serious Illness in the past 2 years (Ref: No)	-	-	-	-
Hypertension	1.119	0.375	-6.287	-4.542**
	(0.889)	(1.117)	(4.531)	(1.950)
Diabetes	1.600	0.111	-6.180	-3.150
	(1.055)	(1.284)	(3.701)	(2.336)
Heart Disease	0.790	0.076	-7.617*	-4.930**
	(0.942)	(1.157)	(3.674)	(1.839)
Stroke or CVD	1.082	0.271	-6.804**	-4.415**
	(0.998)	(1.125)	(3.264)	(1.571)
Bronchitis	0.362	-0.898	-6.627	-4.074**
	(0.858)	(1.139)	(3.948)	(1.907)
Tuberculosis	4.336**	2.070		
	(2.076)	(2.339)		
Cataracts	0.386	-0.209	-7.497*	-4.876**
	(0.869)	(1.074)	(4.231)	(2.057)
Glaucoma	1.544*	1.204	-7.449	-2.106
	(0.913)	(1.195)	(4.438)	(3.724)
Cancer	1.129	-0.961	-8.768	-7.401**
	(1.181)	(1.878)	(5.511)	(3.482)
Prostate Tumor	0.883	0.017	-6.403	-3.474
	(0.846)	(1.090)	(4.430)	(2.430)

Gastric	1.343	0.613	-7.859**	-5.638**
	(0.913)	(1.167)	(3.724)	(2.033)
Parkinson	3.814***	2.219	-2.055	-1.000
	(1.443)	(1.469)	(5.145)	(3.107)
Bedsore	-1.472	-3.763***		
	(1.067)	(1.265)		
Arthritis	0.454	0.038	-8.299*	-5.142*
	(0.851)	(1.008)	(4.721)	(2.650)
Dementia	2.020*	1.624		
	(1.037)	(1.260)		
Epilepsy	-	-	-	-
Cholecystitis	1.200	0.186	-5.344	-2.204
	(0.914)	(1.224)	(4.584)	(2.310)
Blood Disease	0.762	-0.517	-5.610	-3.698
	(0.965)	(1.149)	(4.163)	(2.267)
Chronic Nephritis	-0.278	-1.967	-10.650**	-9.027***
	(0.975)	(1.250)	(4.897)	(3.041)
Galactophore	0.344	0.020	-4.681	-3.386
	(1.120)	(1.362)	(3.037)	(3.182)
Uterine Tumor	1.770*	-0.127	-6.834	-3.747
	(1.058)	(1.133)	(6.602)	(3.395)
Hepatitis	-	-		
Year = 2011	1.655	0.978		
	(1.501)	(1.467)		
Year = 2014	2.974	1.360	0.433	-2.242
	(2.925)	(2.850)	(3.392)	(3.878)
Observations	1,012	1,012	778	778
Number of id	716	716	621	621
Adjusted R-squared	0.144	0.140	0.182	0.188

Note: *** P<0.01 ** P<0.05 * P<0.1; Robust Standard errors clustered in province level are reported in the Parentheses.

Table S9. The impact of CMI on healthcare expenditures among the Chinese elderly (Fixed effects model and excluding those confounding provinces)

Variables	(1) Logarithm Total Medical Expenditure	(2) Logarithm Total OOP Expenditure	(3) Logarithm Inpatient Expenditure	(4) Logarithm Inpatient OOP Expenditure

$treat_{ipt} \times Post_{it}$	0.752**	0.588***	0.588**	0.652**
	(0.328)	(0.197)	(0.257)	(0.251)
Age	0.352	0.422	0.233	0.288
	(0.236)	(0.255)	(0.169)	(0.219)
Female (Ref: Male)	-	-	-	-
Years of education	-	-	-	-
Urban or town (Ref: Rural)	0.194	0.050	0.028	0.113
	(0.303)	(0.271)	(0.545)	(0.509)
Married (Ref: No)	-0.131	0.006	0.813***	0.334
	(0.209)	(0.197)	(0.217)	(0.282)
Number of Children alive	0.004	0.011	-0.143***	-0.267***
	(0.050)	(0.045)	(0.035)	(0.052)
Live alone (Ref: No)	-0.420**	-0.228	-0.225	-0.087
	(0.187)	(0.194)	(0.377)	(0.349)
URBMI (Ref: NCMS)	0.452***	0.198	0.345	0.337
	(0.136)	(0.148)	(0.270)	(0.278)
Logarithm of household income per capita	-0.023	-0.034	0.004	-0.054
	(0.037)	(0.032)	(0.063)	(0.071)
Self-rated Health	0.344***	0.367***	0.129	0.222**
	(0.054)	(0.051)	(0.093)	(0.100)
Number of chronic diseases	-0.707	-0.918	2.003***	1.951***
	(1.245)	(1.285)	(0.660)	(0.567)
Serious Illness in the past 2 years (Ref: No)	2.051***	1.946***	4.312***	3.652***
	(0.183)	(0.155)	(0.224)	(0.208)
Hypertension	1.086	1.311	-1.897**	-1.969***
	(1.288)	(1.292)	(0.767)	(0.626)
Diabetes	1.727	1.668	-1.830*	-1.605
	(1.500)	(1.489)	(0.981)	(0.959)
Heart Disease	0.992	1.425	-1.639**	-1.657**
	(1.319)	(1.382)	(0.634)	(0.611)
Stroke or CVD	0.958	1.026	-1.780**	-1.805**
	(1.241)	(1.289)	(0.712)	(0.662)
Bronchitis	0.900	1.140	-1.630**	-1.433*
	(1.182)	(1.235)	(0.760)	(0.727)
Tuberculosis	0.850	0.809	-2.971***	-3.164**
	(1.441)	(1.461)	(1.000)	(1.098)
Cataracts	0.805	1.122	-2.383***	-2.169***
	(1.253)	(1.340)	(0.725)	(0.629)
Glaucoma	1.721	1.776	-0.402	0.051
	(1.245)	(1.240)	(1.362)	(1.309)
Cancer	2.610	2.174	-1.384	-2.086

	(1.508)	(1.966)	(2.285)	(2.030)
Prostate Tumor	0.799	1.121	-1.816*	-1.633**
	(1.226)	(1.323)	(0.939)	(0.765)
Gastric	1.199	1.527	-1.820**	-1.635**
	(1.277)	(1.290)	(0.782)	(0.763)
Parkinson	1.950	1.955	-0.677	-1.018
	(1.660)	(1.684)	(1.974)	(1.706)
Bedsore	-1.080	-0.777	-1.432	-1.135
	(1.512)	(1.313)	(1.908)	(1.716)
Arthritis	1.233	1.438	-2.324***	-2.217***
	(1.316)	(1.336)	(0.618)	(0.450)
Dementia	-0.630	-0.768	-1.853	-1.673
	(1.656)	(1.471)	(1.286)	(1.189)
Epilepsy	2.319	3.863*		
	(1.365)	(1.874)		
Cholecystitis	0.893	0.841	-1.144	-1.203
	(1.211)	(1.240)	(0.875)	(0.796)
Blood Disease	0.719	0.890	-0.910	-0.897
	(1.240)	(1.262)	(0.945)	(0.965)
Chronic Nephritis	0.083	-0.294	-1.484	-1.658
	(1.373)	(1.321)	(1.175)	(1.090)
Galactophore	-	-		
Uterine Tumor	1.266	1.425	-2.028	-2.128*
	(1.873)	(2.046)	(1.245)	(1.092)
Hepatitis	-0.291	-0.215	-2.154**	-2.508**
	(1.358)	(1.430)	(0.870)	(0.891)
Year = 2011	-1.088	-1.378		
	(0.790)	(0.835)		
Year = 2014	-1.773	-2.515	-0.365	-0.733
	(1.427)	(1.487)	(0.350)	(0.545)
Observations	3,711	3,711	2,474	2,474
Number of id	1,237	1,237	1,237	1,237
Adjusted R-squared	0.156	0.125	0.299	0.284

Note: *** P<0.01 ** P<0.05 * P<0.1; Robust Standard errors clustered in province level are reported in the Parentheses.

Table S10. The impact of CMI on healthcare expenditures among the Chinese elderly (Fixed effects model based on the Propensity score matching)

	(1)	(2)	(3)	(4)
Variables	Logarithm	Logarithm	Logarithm	Logarithm

	Total Medical Expenditure	Total OOP Expenditure	Inpatient Expenditure	Inpatient OOP Expenditure
$treat_{ipt} \times Post_{it}$	0.773**	0.645***	0.581**	0.621**
	(0.317)	(0.180)	(0.229)	(0.241)
Age	0.344	0.389*	0.382**	0.515**
	(0.207)	(0.208)	(0.144)	(0.205)
Female (Ref: Male)	-	-	-	-
Years of education	-	-	-	-
Urban or town (Ref: Rural)	0.106	-0.136	0.191	0.242
	(0.328)	(0.299)	(0.428)	(0.405)
Married (Ref: No)	-0.171	-0.057	0.342	-0.019
	(0.180)	(0.183)	(0.339)	(0.311)
Number of Children alive	-0.005	-0.002	-0.077**	-0.163***
	(0.032)	(0.030)	(0.034)	(0.056)
Live alone (Ref: No)	-0.419**	-0.201	-0.137	-0.007
	(0.171)	(0.185)	(0.318)	(0.298)
URBMI (Ref: NCMS)	0.317**	0.157	0.136	0.113
	(0.134)	(0.119)	(0.247)	(0.259)
Logarithm of household income per capita	-0.017	-0.030	-0.005	-0.068
	(0.035)	(0.031)	(0.058)	(0.069)
Self-rated Health	0.393***	0.412***	0.193*	0.246**
	(0.064)	(0.060)	(0.097)	(0.094)
Number of chronic diseases	1.921***	4.896***	1.818***	1.799***
	(0.375)	(0.277)	(0.561)	(0.506)
Serious Illness in the past 2 years (Ref: No)	1.891***	1.872***	4.411***	3.792***
	(0.179)	(0.132)	(0.195)	(0.199)
Hypertension	-1.531***	-4.499***	-1.734**	-1.836***
	(0.419)	(0.343)	(0.621)	(0.522)
Diabetes	-0.979*	-4.226***	-1.553*	-1.327
	(0.489)	(0.382)	(0.881)	(0.832)
Heart Disease	-1.557***	-4.439***	-1.604***	-1.660***
	(0.377)	(0.283)	(0.559)	(0.545)
Stroke or CVD	-1.635***	-4.775***	-1.476**	-1.554**
	(0.385)	(0.331)	(0.612)	(0.583)
Bronchitis	-1.669***	-4.584***	-1.338**	-1.280**
	(0.416)	(0.311)	(0.601)	(0.577)
Tuberculosis	-2.111**	-5.027***	-2.971***	-2.945***
	(0.930)	(0.705)	(0.922)	(0.990)
Cataracts	-1.810***	-4.706***	-2.253***	-2.054***
	(0.430)	(0.321)	(0.614)	(0.562)

Glaucoma	-1.019**	-4.162***	-1.329	-0.943
	(0.448)	(0.368)	(1.283)	(1.189)
Cancer	-0.125	-3.342***	-0.753	-1.319
	(0.708)	(0.931)	(1.789)	(1.668)
Prostate Tumor	-2.000***	-4.865***	-1.931**	-1.743**
	(0.482)	(0.462)	(0.847)	(0.712)
Gastric	-1.636***	-4.516***	-1.930***	-1.769***
	(0.363)	(0.278)	(0.612)	(0.605)
Parkinson	-0.841	-3.840***	0.145	-0.674
	(0.624)	(0.583)	(1.740)	(1.541)
Bedsore	-6.171***	-8.686***	-2.823	-2.606
	(1.364)	(1.262)	(1.672)	(1.617)
Arthritis	-1.447***	-4.400***	-2.115***	-2.039***
	(0.366)	(0.255)	(0.560)	(0.431)
Dementia	-3.427***	-6.691***	-2.215	-1.986
	(0.777)	(0.579)	(1.344)	(1.276)
Epilepsy	-	-	-	-
Cholecystitis	-1.736***	-4.892***	-1.245*	-1.145*
	(0.399)	(0.344)	(0.679)	(0.628)
Blood Disease	-1.944***	-4.883***	-0.942	-1.047
	(0.459)	(0.394)	(0.792)	(0.824)
Chronic Nephritis	-2.221***	-5.332***	-0.463	-0.520
	(0.585)	(0.561)	(1.053)	(1.000)
Galactophore	-1.520	-4.625***	-0.971	-1.466
	(1.384)	(1.464)	(1.223)	(1.245)
Uterine Tumor	-1.053	-4.069***	-1.607	-1.433
	(0.999)	(0.986)	(1.508)	(1.581)
Hepatitis	-2.410***	-5.247***	-1.234	-1.438*
	(0.513)	(0.604)	(0.846)	(0.771)
Year = 2011	-1.080	-1.323*		
	(0.703)	(0.692)		
Year = 2014	-1.737	-2.387*	-0.790**	-1.347**
	(1.252)	(1.218)	(0.347)	(0.548)
Observations	4,605	4,605	3,070	3,070
Number of id	1,535	1,535	1,535	1,535
Adjusted R-squared	0.150	0.126	0.315	0.295

Note: *** P<0.01 ** P<0.05 * P<0.1; Robust Standard errors clustered in province level are reported in the Parentheses.
